# Plasma Metabolites Related to Peripheral and Hepatic Insulin Sensitivity Are Not Directly Linked to Gut Microbiota Composition

**DOI:** 10.3390/nu12082308

**Published:** 2020-07-31

**Authors:** Annefleur M. Koopen, Nicolien C. de Clercq, Moritz V. Warmbrunn, Hilde Herrema, Mark Davids, Pieter F. de Groot, Ruud S. Kootte, Kristien E. C. Bouter, Max Nieuwdorp, Albert K. Groen, Andrei Prodan

**Affiliations:** 1 Department of Internal and Vascular Medicine, Amsterdam University Medical Centers, Location AMC, Meibergdreef 9, 1105 AZ Amsterdam, The Netherlands; m.v.warmbrunn@amsterdamumc.nl (M.V.W.); h.j.herrema@amsterdamumc.nl (H.H.); m.davids@amsterdamumc.nl (M.D.); p.f.degroot@amsterdamumc.nl (P.F.d.G.); r.s.kootte@amsterdamumc.nl (R.S.K.); k.e.bouter@amsterdamumc.nl (K.E.C.B.); m.nieuwdorp@amsterdamumc.nl (M.N.); a.k.groen@amsterdamumc.nl (A.K.G.); a.prodan@amsterdamumc.nl (A.P.); 2 Wallenberg Laboratory, University of Gothenburg, Bruna Stråket 16, SE-413 45 Göteborg, Sweden; 3 Department of Laboratory Medicine, University of Groningen, University Medical Center, Grote Kruisstraat 2/1, 9712 CP Groningen, The Netherlands

**Keywords:** gut microbiota, metabolic syndrome, diabetes, insulin sensitivity, plasma metabolites

## Abstract

Plasma metabolites affect a range of metabolic functions in humans, including insulin sensitivity (IS). A subset of these plasma metabolites is modified by the gut microbiota. To identify potential microbial–metabolite pathways involved in IS, we investigated the link between plasma metabolites, gut microbiota composition, and IS, using the gold-standard for peripheral and hepatic IS measurement in a group of participants with metabolic syndrome (MetSyn). In a cross-sectional study with 115 MetSyn participants, fasting plasma samples were collected for untargeted metabolomics analysis and fecal samples for 16S rRNA gene amplicon sequencing. A two-step hyperinsulinemic euglycemic clamp was performed to assess peripheral and hepatic IS. Collected data were integrated and potential interdependence between metabolites, gut microbiota, and IS was analyzed using machine learning prediction models. Plasma metabolites explained 13.2% and 16.7% of variance in peripheral and hepatic IS, respectively. Fecal microbiota composition explained 4.2% of variance in peripheral IS and was not related to hepatic IS. Although metabolites could partially explain the variances in IS, the top metabolites related to peripheral and hepatic IS did not significantly correlate with gut microbiota composition (both on taxonomical level and alpha-diversity). However, all plasma metabolites could explain 18.5% of the variance in microbial alpha-diversity (Shannon); the top 20 metabolites could even explain 44.5% of gut microbial alpha-diversity. In conclusion, plasma metabolites could partially explain the variance in peripheral and hepatic IS; however, these metabolites were not directly linked to the gut microbiota composition, underscoring the intricate relation between plasma metabolites, the gut microbiota, and IS in MetSyn

## 1. Introduction

The current global obesity pandemic has resulted in a dramatic increase in the prevalence of metabolic syndrome (MetSyn) [1]. Although the presence of MetSyn is highly predictive of new-onset diabetes mellitus type 2 (DM2), not all participants meeting the criteria for MetSyn develop DM2 [2]. Research focusing on the mechanisms behind insulin resistance is necessary to better understand the complex pathophysiology of insulin resistance in MetSyn and to ultimately develop new therapies.

In the past decade, both animal [3,4] and human [5,6,7] studies have recognized microbiota-derived metabolites—such as short chain fatty acids (SCFA), 4-cresol, and imidazole propionate—as potential drivers of insulin resistance in MetSyn and DM2 [2,8,9,10,11]. However, many questions remain. First of all, the majority of microbiota-derived metabolites and their potential influence on human metabolism have yet to be identified. A mouse model has shown that only 10% of circulating plasma metabolites are derived from the gut microbiota [12] addressing one of the major challenges in identifying key microbiota–metabolite pathways in the development of insulin resistance. Secondly, to identify microbiota-derived metabolites relevant for the development of insulin resistance, researchers often conduct univariate analysis, correlating plasma metabolite levels with abundances of gut microbial taxa. However, correlation-based analyses often have low statistical value with regard to microbiota–metabolite relationships [13]. A machine learning approach may therefore be a more robust method to unravel microbiota–metabolite interactions. Finally, studies assessing the link between plasma metabolites, gut microbiota, and insulin resistance have used either the homeostasis model assessment (HOMA) index [14], oral glucose tolerance test (OGTT) [2], or fasting hemoglobin A1 (HbA1c%) [15] as markers of insulin resistance. However, insulin resistance in MetSyn and DM2 is characterized by elevated basal endogenous glucose production (EGP), impaired suppression of endogenous glucose production (EGPsupp, a surrogate marker for hepatic IS), and reduced peripheral insulin-stimulated glucose uptake [16]. To unravel the complex mechanisms through which the gut microbiota and its derived metabolites are involved in the pathophysiology of insulin resistance, it is therefore essential to examine hepatic and peripheral IS. The hyperglycemic clamp technique developed by DeFronzo et al. is the current gold standard method to assess EGPsupp and the rate of glucose disappearance (Rd), reflecting hepatic and peripheral insulin resistance, respectively [17,18].

This is the first study that used a machine learning model approach to assess the complex relation between peripheral and hepatic insulin sensitivity (IS; measured by hyperinsulinemic euglycemic clamp), plasma metabolites, and gut microbiota composition in a large group of 115 treatment-naïve MetSyn participants. The aim of this cross-sectional study was to ultimately identify microbial-metabolite pathways involved in variations of IS. We hypothesized that IS is interrelated with gut microbiome composition and plasma metabolites.

## 2. Materials and Methods

### 2.1. Study Design and Participants

We conducted a cross-sectional study on human participants with MetSyn in compliance with the principles of the declaration of Helsinki. All study procedures were approved by the Academic Medical Center ethics committee, location AMC and all patients provided written, informed voluntary consent. Data collection took place between January 2015 and February 2019.

In order to be eligible, all Caucasian male and postmenopausal female participants had to meet three or more criteria of MetSyn:

- fasting plasma glucose ≥ 5.6 mmol/L
- triglycerides ≥ 1.7 mmol/L
- waist-circumference ≥ 102 cm for men and ≥ 88 cm for women
- high-density lipoprotein (HDL-) cholesterol ≤ 1.04 mmol/L
- blood pressure ≥ 130/85 mmHg.

Furthermore, all participants had a body mass index (BMI) > 28 kg/m^2^, were treatment-naïve and otherwise healthy. Main exclusion criteria were a history of cardiovascular events, cholecystectomy, and use of any type of medication. Complete in- and exclusion criteria have been published previously [19].

### 2.2. Outcomes

#### 2.2.1. Metabolic Profiling

**Figure 1:**
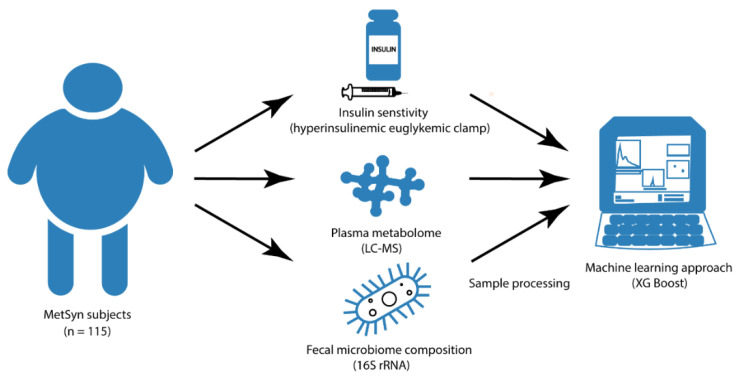
Study design. Insulin sensitivity, plasma metabolites, and fecal microbiota composition were measured in participants with MetSyn (n = 115). Machine learning prediction models were used to evaluate relationships between fecal microbiome composition, plasma metabolites, and insulin sensitivity.

Fasting EDTA plasma samples were taken before the hyperinsulinemic euglycemic clamp (Figure 1). Metabolon (Durham, NC, USA) performed untargeted metabolomic profiling using ultra high performance liquid chromatography coupled to tandem mass spectrometry (UPLC-MS/MS), as previously described [8]. At total of 935 annotated and 236 unannotated plasma metabolites where measured. Raw data was normalized to account for inter-day measurement differences. Then, data were rescaled so that the median level for each metabolite across all samples was equal to 1. Missing values, generally due to the metabolite levels falling below the limit of detection, were then imputed with half the minimum observed value for the respective metabolite across all samples.

#### 2.2.2. Hyperinsulinemic Euglycemic Clamp

A two-step hyperinsulinemic euglycemic clamp with stable isotopes was performed in all participants to assess insulin-mediated suppression of endogenous glucose production (EGPsupp) and rate of glucose disappearance (Rd) [20]. Briefly, baseline blood samples were drawn after an overnight fast to determine background isotope enrichment. A primed continuous infusion of [6,6-2H2] glucose was started and continued until the end of the test (prime: 11 μmol kg^−1^; continuous: 0.11 μmol kg^−1^min^−1^). After two hours of equilibration, infusion of insulin (Actrapid; Novo Nordisk Farma B.V., Alphen aan den Rijn, The Netherlands) was started at a rate of 20 mU m^−2^ min^−1^. Venous glucose concentrations were measured every 10 min and 20% glucose enriched with 1% [6,6-2H2] glucose was infused at a variable rate to maintain plasma glucose at 5 mmol/L. After two hours, the insulin infusion rate was increased to 60 mU m^−2^ min^−1^ for an additional two hours. At the end of both steps, five repetitive arterialized venous blood samples were drawn with an interval of 5 min to determine glucose enrichments and hormones. Glucose fluxes were calculated using the modified forms of the Steele equations for (non-)steady state measurements [21].

#### 2.2.3. Fecal Microbiota and Dietary Intake

Fresh morning stool samples were taken on the day of the hyperinsulinemic euglycemic clamp (Figure 1). Participants were allowed to continue their own diet; three days prior to the study visit, participants were asked to keep an online nutritional diary (www.voedingscentrum.nl) to monitor nutritional intake including. This food frequency questionnaire assessed the daily amount of caloric intake combined with portion and frequency of micro- and macronutrients, including fat, carbohydrates, proteins, and fibers.

DNA was extracted from fecal material using a repeated bead beating protocol [22]. DNA was purified using Maxwell RSC Whole Blood DNA Kit. 16S rRNA gene amplicons were generated using a single step PCR protocol targeting the V3-V4 region [23]. PCR products were purified using Ampure XP beads and purified products were equimolar pooled. The libraries were sequenced using a MiSeq platform using V3 chemistry with 2 × 251 cycles.

Forward and reverse reads were truncated to 240 and 210 bases respectively and merged using USEARCH [24]. Merged reads that did not pass the Illumina chastity filter, had an expected error rate higher than 2, or were shorter than 380 bases were removed. Amplicon sequence variants (ASVs) were inferred for each sample individually from sequences with a minimum abundance of four reads [24]. Unfiltered reads were then mapped against the joint ASV set to determine per sample ASV abundances. Taxonomy was assigned using the RDP classifier [25] and SILVA [26] 16S ribosomal database V132. Microbial counts were rarefied to 30,000 counts per sample.

#### 2.2.4. Machine Learning Models and Statistical Analysis

The XGBoost (v. 0.90) implementation of gradient boosted trees was used in prediction models structured in a nested-cross validation system to prevent overfitting and ensure robustness of results (Supplemental Figure S1). The models were structured according to an iterative flow, as follows. In each iteration, the dataset was randomly split into a test set (20% of participants) and a training set (80% of participants). Only within the training set, five-fold cross-validation (CV) was performed to fit and optimize the model hyperparameters. Finally, the optimized model was then tested on the examples in the test set (never used thus far). Explained variance (% variance in the true outcome y explained by the outcome values predicted by the model, $\hat{y}$ see formula (1) below) and the ranked feature importance list were recorded for each iteration.

$$Explainedvariance\left(y,\hat{y}\right)=1-\frac{Var\left\{y-\hat{y}\right\}}{Var\left\{y\right\}}*100\%$$

Explained variance and feature importances were recorded for each iteration and were averaged across 200 iterations to produce the final model results. If the explained variance was negative, we concluded that the model did not have any predictive power, e.g., no relation between the two outcome measures. Furthermore, two random variables were added to the predictor data during each iteration to serve as a benchmark for feature importance interpretation (i.e., any feature ranked lower than one of the random variables was regarded as having no predictive power). The models were implemented in Python (v. 3.7.4) using numpy (v. 1.16.4), pandas (v. 0.25.1), and scikit-learn (v. 0.21.2) packages. Statistical analyses and visualizations were performed in R (v. 3.6.2) using the ggplot2 package. Non-parametric tests were used to assess correlations (Spearmans’s rho). The Benjamini–Hochberg procedure was used to correct *p*-values for multiple comparisons.

## 3. Results

### 3.1. Participants Characteristics

We included 115 participants with MetSyn with a mean BMI of 34.2 ± 3.9 (Table 1). EGPsupp, assessed during the first step of the clamp, was 68.7 ± 16.0%. Rd in the second step of the clamp was 33.3 ± 13.2 μmol kg^−1^min^−1^. The mean caloric self-reported intake was 1936 ± 449 kcal/day.

### 3.2. Plasma Metabolites and Insulin Sensitivity

**Figure 2:**
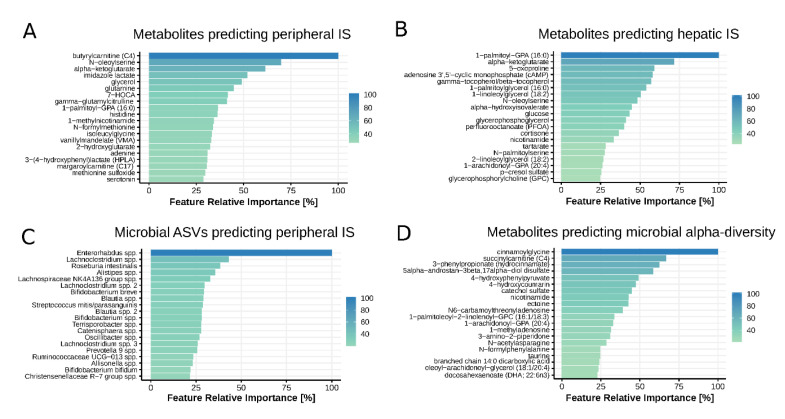
Feature importance plots. Overview of top 20 features (metabolites or microbial ASVs) explaining variance in insulin sensitivity and gut microbial alpha-diversity best. (**A**) Metabolites explaining variance in peripheral insulin sensitivity, (**B**) Metabolites explaining variance in hepatic insulin sensitivity, (**C**) Microbial ASVs explaining variance in peripheral insulin sensitivity, and (**D**) Metabolites explaining variance in gut microbial alpha-diversity. ASV: amplicon sequence variant.

Plasma metabolites explained 13.2% and 16.7% of the variance in peripheral and hepatic IS, respectively. Figure 2A,B present feature importance plots of the top 20 plasma metabolites explaining variance in peripheral and hepatic IS best. These plots indicate the relative importance of each metabolite in explaining variances in IS. Correlations (Spearman’s rho) between the top 20 metabolites, including corresponding metabolic pathways, are presented in Supplemental Tables S1 and S2.

The majority of the top 20 plasma metabolites explaining peripheral and hepatic IS belonged to the lipid and amino acid superfamilies, in particular intermediates of fatty acid and amino acid metabolism. The most important metabolite explaining variance in peripheral IS, was butyrylcarnitine (C4) (rho = −0.35, *p* = 0.0011), a member of the acylcarnitines. Butyrylcarnitine levels reflect concentrations of tissue butyryl-CoA, a biomarker of obstructed fatty acid beta-oxidation and subsequent depletion of tricarboxylic acid (TCA)-cycle intermediates [15]. Notably, the TCA cycle intermediate alpha-ketoglutarate was third and second most important metabolite explaining variance in peripheral and hepatic IS, respectively. Four metabolites were among the top 20 metabolites for both peripheral and hepatic IS: N-oleoylserine, alpha-ketoglutarate, histidine, and 1-palmitoyl-GPA (16:0). Of note, these four plasma metabolites all correlated significantly with both Rd and EGPsupp (*p* < 0.05).

### 3.3. Gut Microbiota and Insulin Sensitivity

Next, we used the machine learning model to assess the relation between gut microbiota composition (ASV relative abundances), microbial alpha-diversity, and IS. Fecal microbiota composition explained 4.2% of peripheral IS variance, but could not explain variance in hepatic IS. The top 20 microbes explaining variance in peripheral IS are presented in Figure 2C, whereas the correlations are shown in Supplemental Table S3. Furthermore, there was no significant correlation between α-diversity (Shannon index) and peripheral or hepatic IS (Spearman rho *p* = 0.91 and *p* = 0.16, respectively). There was no significant effect of dietary factors (total daily caloric intake, carbohydrate, fat, protein, and fiber intake) on either gut microbial alpha-diversity (all Spearman rho correlations with Shannon index *p* > 0.05) or beta-diversity (all *p*-values of PERMANOVA on Bray–Curtis distances > 0.05), thought reliability of dietary data is questionable.

### 3.4. Interrelation between Plasma Metabolites, Gut Microbiota, and Insulin Sensitivity

To further explore the relationship between metabolites, gut microbiota composition, and IS, we performed Spearman’s rank correlations between the top 20 metabolite explaining variance in IS and the gut microbiota. After correction for multiple comparisons, there were no significant correlations between the top 20 metabolites explaining variance in either peripheral or hepatic IS and gut microbial ASVs. Furthermore, there was no correlation between these top 20 metabolites and microbial alpha-diversity (Shannon index).

### 3.5. Plasma Metabolites and Gut Microbiota Alpha-Diversity

Although we did not find a direct relation between metabolites explaining variance in IS and the gut microbiota, there was a strong relation between all fasting plasma metabolites and microbial alpha-diversity (Shannon index). A prediction model based on plasma metabolites could explain 18.5% of the variance in microbial alpha-diversity. A refined model using only the 20 most predictive metabolites explained 44.7% of variance in gut microbial alpha-diversity (Figure 2D).

## 4. Discussion

**Figure 3:**
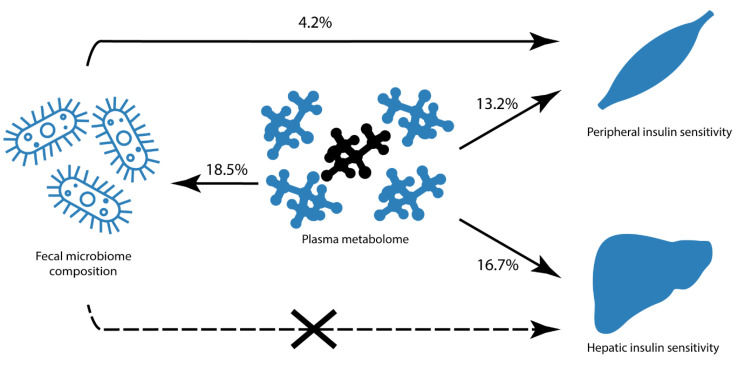
Graphical depiction of relationships between biological dimensions. Associations are expressed in percentage of explained variance (where at least some of the variance in the phenotype could be explained by the model). The machine learning model was able to explain a relatively high variance of hepatic insulin sensitivity (16.7%) and peripheral insulin sensitivity (13.2%). Models using gut microbiome composition (on taxonomical level) to predict insulin sensitivity only found an explained variance for peripheral IS (4.2%) and no link with hepatic insulin sensitivity. Plasma metabolites could explain 18.5% of the variance in gut microbial alpha-diversity and even 44.7% when using the top 20 metabolites.

In the present study, we confirm the link between fasting plasma metabolites and IS, using the gold standard method for IS measurement, in a large treatment-naïve and relatively healthy group of participants with MetSyn. The large dynamic range in both peripheral and hepatic IS in these participants enabled a sensitive survey of the association between these parameters and the gut microbiota. Surprisingly, the explained variance between the gut microbiota composition and peripheral IS was only 4.2%, whereas no association was observed with hepatic IS (Figure 3). Moreover, although plasma metabolites overall showed a strong link with gut microbiota diversity in our cross-sectional cohort, specific plasma metabolites that were found to explain variance in IS were not directly related to gut microbiota composition, underscoring the complex relation between circulating metabolites, gut microbiota, and IS (Figure 3).

### 4.1. Plasma Metabolites and Insulin Sensitivity

Multiple smaller studies have found associations between human participants with MetSyn and fasting plasma metabolites involved in lipid (phospholipids, sphingomyelins, and triglycerides), amino acid (branched chain amino acids, glycine, and glutamine), and carbohydrate (glucose and fructose) metabolism [7]. Therefore, it was not surprising to find that the majority of the top 20 metabolites explaining variance in peripheral and hepatic IS were either directly or indirectly involved in lipid or amino acid metabolism (Supplemental Tables S1 and S2). Excessive lipid exposure and consequent accumulation of ectopic triglycerides affect multiple metabolic pathways leading to impaired glucose tolerance [27,28]. One common theory states that peripheral insulin resistance is caused by dietary fat overload inducing an accumulation of triacylglycerol in peripheral non-adipose tissues [29,30]. In short, accumulation of lipids in muscles and liver initiates insulin resistance, as ectopic lipids inhibit the actions of insulin such as glucose uptake and inhibition of lipolysis. This pathophysiological model proposes that a diminished activity of the mitochondrial enzyme carnitine palmitoyltransferase-1 (CPT-1)—which catalyzes the conversion of long-chain acyl-CoA into long-chain acylcarnitine—leads to decreased mitochondrial fatty acid oxidation, thus redirecting free fatty acids towards triglyceride synthesis [31]. With respect to fatty acid oxidation, plasma butyrylcarnitine (C4), which was the most important metabolite explaining variance in peripheral IS, can be derived from either fatty acid or amino acid degradation [32,33,34]. This metabolite indirectly reflects concentrations of alpha-ketoglutarate, an intermediate metabolite of the TCA-cycle [15], which was also an important metabolite explaining variance in peripheral and hepatic IS in our study.

Plasma metabolites explaining variance in hepatic IS where also involved in fatty acid signaling, especially the top metabolite, 1-palmitoyl-GPA or lysophosphatidylcholine (rho = 0.522, *p* = < 0.001). This bioactive lipid mediator is involved in numerous cellular lipid signaling processes [35,36], including enhancement of glucose-dependent insulin secretion from beta-cells [37]. Our findings are in line with those from an animal study that reported decreased levels of plasma lysophohsphatidylcholine in obese and diabetic mice [35].

### 4.2. Gut Microbiota Composition and Insulin Sensitivity

Large metagenome-wide association studies showed that both gut microbiota composition and alpha diversity differ between healthy, obese, and (pre) DM2 individuals [10,38,39]. However, the exact pathophysiological role of the microbiota in IS in humans is still extensively being studied and debated, and evidence regarding causality is scarce [40,41]. In the present study, we observed that in a group of MetSyn participants, gut microbiota composition explained 4.2% of the variance in peripheral IS, but none of the variance in hepatic IS, which is in line with association studies and with our previous research showing an effect of fecal microbiota transplantation on peripheral IS, but not on hepatic IS [5,19,41]. In this regard, an ASV belonging to the genus *Enterorhabdus* was the most important specie explaining variance in peripheral IS is (rho = 0.0272, *p* = 0.038). This genus has been previously identified exclusively in lean mice and not present in obese mice [42,43]. Moreover, the majority of the top 20 bacteria belong to the *Lachnospiraceae* family, including 4 out of 6 top bacteria, correlating negative with IS. This is in concordance with previous studies; the introduction of a *Lachnospiraceae* strain in germ free mice contributed to the development of DM2 and in several human studies the *Lachnospiraceae* family is associated with impaired glucose tolerance and DM2 [10,44,45,46].

### 4.3. Interrelation of Plasma Metabolites with Gut Microbiota and Insulin Sensitivity

The absence of a direct link between the metabolites related to peripheral and hepatic IS and gut microbiota composition stresses the challenges observed in this field of research, both on a technical and biological level. The first crucial challenge is that most of the gut microbes modulating levels of plasma metabolites remain unknown, as are the mechanisms by which these microbes exert their action [13]. Although for some metabolites—such as trimethylamine N-oxide, branched chain amino acids, and butyrate—the bacterial species contribute to their variability are known, this is not always the case for dietary derived metabolites that can be synthesized or degraded by many different microbial taxa [13]. Ideally, in order to untangle the direct and indirect effects of individual species on plasma metabolite concentrations, a reductionist approach should be pursued by first applying mechanistic intervention studies to dissect causality, subsequently followed by single species interventions (as we previously published) [47]. Secondly, plasma metabolites in peripheral blood may not be able to completely explain the intricate relation between whole body IS and gut microbiota composition, as some essential microbial-derived metabolites with a short half-life cannot be found in peripheral circulation. These metabolites could only be measured in samples from the portal vein (that carries blood from the gut to the liver), which was not sampled due to ethical concerns posed by the invasive and hazardous procedure that would be required for collection.

However, we found a strong link between gut microbiota diversity and plasma metabolites, with a refined model using the top 20 metabolites explaining 44.7% of variance in gut microbial alpha-diversity. Interestingly, these results closely resemble those from the recently published work of Wilmanski et al. [48]. In this study, they used LASSO machine learning models to predict gut microbial alpha-diversity from blood metabolites in a cohort of 399 healthy individuals without specific determination of (stable isotope based) glucose fluxes. Three of the most predictive metabolites in our study were also among the metabolites found by Wilmanski et al., including plasma 5α-androstan-3β-17α-diol disulfate, cinnamoylglycine, and 3-phenylpropionate (Figure 2D). Together, the combined findings of the present study and of Wilmanski et al. validate the link between plasma metabolites and gut microbial alpha-diversity.

### 4.4. Limitations

This study has several limitations. First, we used an untargeted HPLC-MS approach to explore plasma metabolites profiles in relation to (stable isotope-based) hepatic and peripheral IS. This analytical method only provides relative metabolite levels rather than absolute concentrations, yet has greater sensitivity and is less biased compared to a targeted approach. Although we used the gold standard stable isotope technique to study glucose fluxes in humans, more dynamic measurements using plasma samples taken at several time points from each individual could provide information on plasma metabolite turnover. In addition to this, we used 16S rRNA gene amplicon sequencing to evaluate gut microbiota composition. This is limited by the discriminating power of the targeted hypervariable region (i.e., V3-V4), and thus cannot differentiate species that have identical sequences in this region. Evaluating gut microbiota using shotgun sequencing could have provided a direct link between specific microbes and their function in relation to plasma metabolites. However, metagenomics analysis is more expensive and the potential to produce certain metabolites does not imply constant production, as research with SCFA has demonstrated [49]. Also, we did not monitor intestinal motility and stool frequency, factors known to influence fecal microbiota composition [50].

Furthermore, this study is potentially biased by the unequal sex distribution of the study participants—e.g., 90% of the study participants was male—which is known to influences gut microbiota composition [51,52]. Also, dietary intake was self-reported, which is known to be unreliable and might explain the low caloric intake.

Finally, our study was also limited by its cross-sectional nature. In order to identify and validate metabolites as potential biomarkers for the development of DM2, a longitudinal study with multiple sampling time points is needed.

## 5. Conclusions

The aim of this cross-sectional study was to integrate plasma metabolites, gut microbiota composition, and hyperinsulinemic clamp data to ultimately identify specific microbial-metabolite pathways that drive variations in human IS in treatment-naïve MetSyn participants. The lack of such a direct connection between plasma metabolites, IS, and gut microbiota composition stresses the complexity of the microbial-metabolite signaling when using cross-sectional studies. Future studies should have a longitudinal design and take into account factors affecting microbial-metabolite pathways, such as different classes of signaling molecules (e.g., proteins), to better understand the intricate pathophysiology of insulin resistance.

## Figures and Tables

**Table 1:** Baseline characteristics, expressed as mean ± SEM or median [IQR].

	Total Group (n = 115)
Male gender (%)	90
Age (years)	55.9 ± 8.1
Weight (kg)	111.0 ± 15.5
BMI (kg/m2)	34.2 ± 3.9
Blood pressure: systolic (mmHg)	143 ± 18
Blood pressure: diastolic (mmHg)	89 ± 11
Fasting glucose (mmol/L)	5.8 ± 0.6
Insulin (pmol/L)	112 ± 51
HOMA-IR	4.0 ± 2.0
Cholesterol: total (mmol/L)	5.5 ± 1.1
Cholesterol: HDL (mmol/L)	1.2 ± 0.3
Cholesterol: LDL (mmol/L)	3.6 ± 0.9
Cholesterol: triglycerides (mmol/L)	1.5 ± 0.6
ALT (U/L)	31 (24–39)
CRP (mg/mL)	2.0 (1.1–4.2)
Rd (μmol kg^−1^min^−1^)	33.3 ± 13.2
EGP suppression (%)	68.7 ± 16.0
Energy intake (kcal/day)	1936 ± 449
Fat intake (gram/day)	73 ± 22
Carbohydrate intake (gram/day)	199 ± 64
Protein intake (gram/day)	88 ± 19
Fiber intake (gram/day)	18 ± 5

BMI: Body Mass Index; HOMA–IR: Homeostatic Model Assessment for Insulin Resistance; HDL: High-Density Lipoprotein; LDL: Low-Density Lipoprotein; ALT: Alanine Transaminase; CRP: C-Reactive Protein; Rd: Rate of glucose disappearance; EGP: Endogenous Glucose Production.

## Supplementary Materials

The following are available online at https://www.mdpi.com/2072-6643/12/8/2308/s1, Figure S1: XGBoost model diagram, Table S1: Correlation top 20 plasma metabolites and peripheral IS, Table S2: Correlation top 20 plasma metabolites and hepatic IS, Table S3: Correlation top 20 microbes and peripheral IS.
