# Supplementary material for: Plasma Metabolites Related to Peripheral and Hepatic Insulin Sensitivity Are Not Directly Linked to Gut Microbiota Composition
Source: Nutrients. 2020 Jul 31;12(8):2308. doi: 10.3390/nu12082308 (PMC7469041; doi:10.3390/nu12082308)
Supplement: Supplementary file 1 [file nutrients-12-02308-s001.pdf]

Supplementary Materials

Plasma Metabolites Related to Peripheral and Hepatic Insulin Sensitivity are not Directly Linked to Gut Microbiota Composition

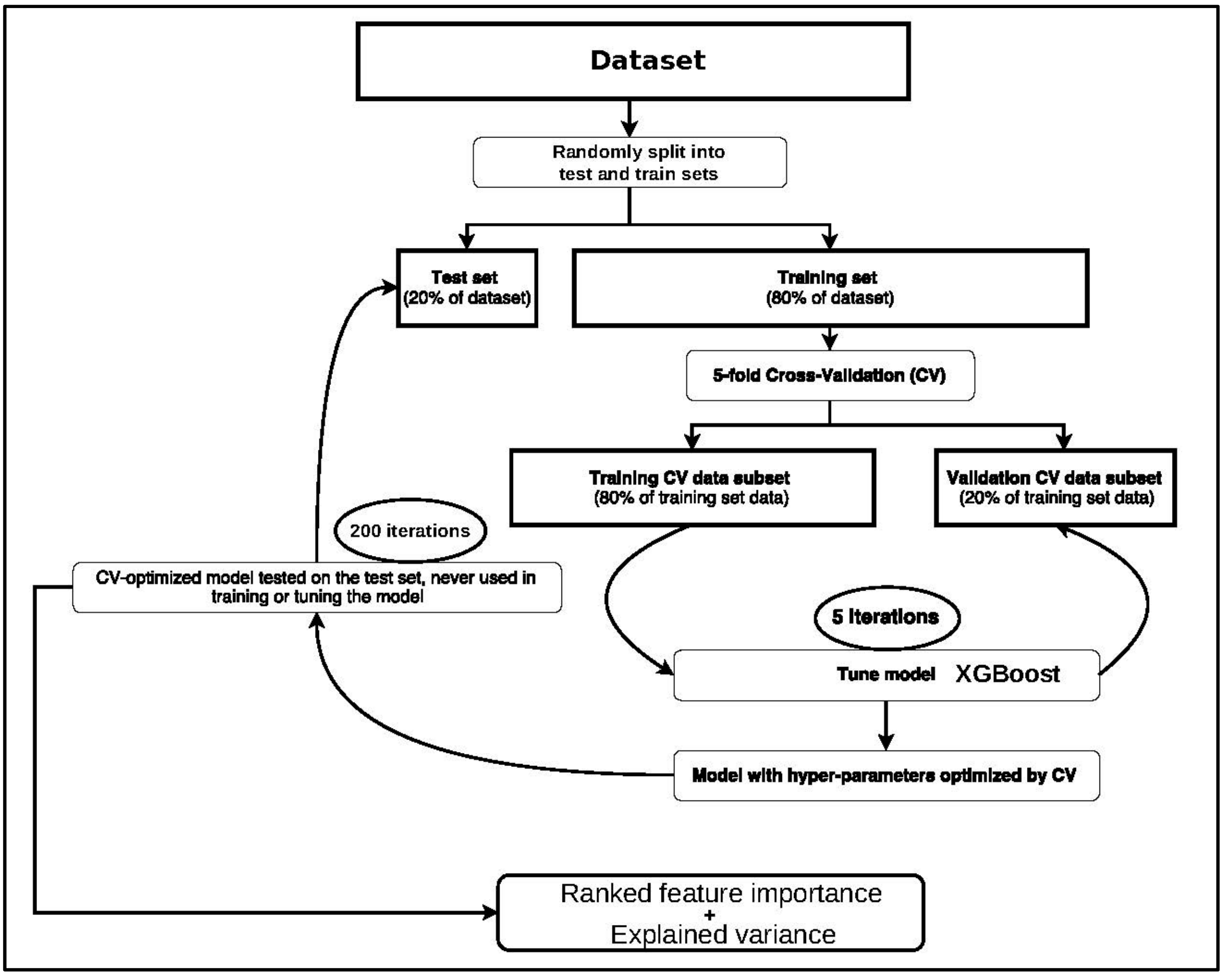

Supplemental Figure 1. XGBoost model diagram

Importance	Feature	Sub pathway	Super pathway	rho	P value	P value adj FDR
**100**	butyrylcarnitine (C4)	Fatty Acid Metabolism (also BCAA Metabolism)	Lipid	-0,35	1,15E-04	1,15E-03
**69,7**	N-oleoylserine	Endocannabinoid	Lipid	0,32	4,54E-04	1,51E-03
**61,37**	alpha-ketoglutarate	TCA Cycle	Energy	-0,33	3,92E-04	1,51E-03
**51,97**	imidazole lactate	Histidine Metabolism	Amino Acid	0,31	8,05E-04	2,30E-03
**48,97**	glycerol	Glycerolipid Metabolism	Lipid	-0,32	4,12E-04	1,51E-03
**44,69**	glutamine	Glutamate Metabolism	Amino Acid	0,21	2,31E-02	3,56E-02
**41,57**	7-HOCA	Sterol	Lipid	-0,33	3,31E-04	1,51E-03
**41,07**	gamma-glutamylcitrulline	Gamma-glutamyl Amino Acid	Peptide	0,24	9,04E-03	1,64E-02
**36,32**	1-palmitoyl-GPA (16:0)	Lysophospholipid	Lipid	0,37	5,41E-05	1,08E-03
**36,04**	histidine	Histidine Metabolism	Amino Acid	0,24	1,00E-02	1,67E-02
**34,22**	1-methylnicotinamide	Nicotinate and Nicotinamide Metabolism	Cofactors and Vitamins	0,26	5,67E-03	1,42E-02
**33,67**	N-formylmethionine	Methionine, Cysteine, SAM and Taurine Metabolism	Amino Acid	-0,06	5,55E-01	5,55E-01
**33,19**	isoleucylglycine	Dipeptide	Peptide	0,19	4,68E-02	5,78E-02
**32,81**	vanillylmandelate (VMA)	Tyrosine Metabolism	Amino Acid	-0,25	7,73E-03	1,55E-02
**32,27**	2-hydroxyglutarate	Fatty Acid, Dicarboxylate	Lipid	0,18	4,92E-02	5,78E-02
**30,94**	adenine	Purine Metabolism, Adenine containing	Nucleotide	-0,16	8,47E-02	9,41E-02
**30,78**	3-(4-hydroxyphenyl) lactate (HPLA)	Tyrosine Metabolism	Amino Acid	-0,2	3,03E-02	4,04E-02
**30,56**	margaroylcarnitine (C17)	Fatty Acid Metabolism (Acyl Carnitine, Long Chain Saturated)	Lipid	0,2	2,98E-02	4,04E-02
**29,66**	methionine sulfoxide	Methionine, Cysteine, SAM and Taurine Metabolism	Amino Acid	0,25	6,91E-03	1,53E-02
**28,81**	serotonin	Tryptophan Metabolism	Amino Acid	0,13	1,65E-01	1,74E-01

Supplemental table 1. Correlation top 20 plasma metabolites and peripheral IS

Importance	Feature	Sub pathway	Super pathway	rho	P value	P value adj FDR
**100**	1-palmitoyl-GPA (16:0)	Lysophospholipid	Lipid	0,52	2,09E-09	4,19E-08
**71,58**	alpha-ketoglutarate	TCA Cycle	Energy	-0,39	1,84E-05	3,75E-05
**59,17**	5-oxoproline	Glutathione Metabolism	Amino Acid	0,39	1,88E-05	3,75E-05
**58,03**	adenosine 3',5'-cyclic monophosphate (cAMP)	Purine Metabolism, Adenine containing	Nucleotide	0,41	4,81E-06	1,38E-05
**56,99**	gamma-tocopherol/beta-tocopherol	Tocopherol Metabolism	Cofactors and Vitamins	0,23	1,16E-02	1,54E-02
**53,78**	1-palmitoylglycerol (16:0)	Monoacylglycerol	Lipid	0,42	2,59E-06	8,64E-06
**50,59**	1-linoleoylglycerol (18:2)	Monoacylglycerol	Lipid	0,45	4,62E-07	3,08E-06
**48,23**	N-oleoylserine	Endocannabinoid	Lipid	0,44	8,70E-07	4,35E-06
**44,72**	alpha-hydroxyisovalerate	Leucine, Isoleucine and Valine Metabolism	Amino Acid	-0,09	3,26E-01	3,62E-01
**43,14**	glucose	Glycolysis, Gluconeogenesis, and Pyruvate Metabolism	Carbohydrate	-0,28	2,06E-03	3,16E-03
**40,91**	glycerophosphoglycerol	Glycerolipid Metabolism	Lipid	-0,32	5,04E-04	8,40E-04
**39,89**	perfluorooctanoate (PFOA)	Chemical	Xenobiotics	-0,26	4,62E-03	6,60E-03
**36,48**	cortisone	Corticosteroids	Lipid	-0,39	2,06E-05	3,75E-05
**33,36**	nicotinamide	Nicotinate and Nicotinamide Metabolism	Cofactors and Vitamins	-0,08	3,76E-01	3,96E-01
**28,2**	tartarate	Food Component/Plant	Xenobiotics	0,04	6,47E-01	6,47E-01
**27,7**	N-palmitoylserine	Endocannabinoid	Lipid	0,21	2,26E-02	2,82E-02
**26,72**	2-linoleoylglycerol (18:2)	Monoacylglycerol	Lipid	0,42	2,42E-06	8,64E-06
**26,13**	1-arachidonoyl-GPA (20:4)	Lysophospholipid	Lipid	0,49	1,97E-08	1,97E-07
**25,44**	p-cresol sulfate	Benzoate Metabolism	Xenobiotics	0,14	1,37E-01	1,61E-01
**24,75**	glycerophosphorylcholine (GPC)	Phospholipid Metabolism	Lipid	0,41	6,68E-06	1,67E-05

Supplemental table 2. Correlation top 20 plasma metabolites and hepatic IS

Importance	Feature	Phylum	Class	Order	Family	Genus	rho	p_val	p_val_adj
100	Enterorhabdus spp.	Actinobacteria	Coriobacteriia	Coriobacteriales	Eggerthellaceae	Enterorhabdus	0,27	3,84E-03	1,29E-02
43,11	Lachnoclostridium spp.	Firmicutes	Clostridia	Clostridiales	Lachnospiraceae	Lachnoclostridium	-0,27	3,86E-03	1,29E-02
38,23	Roseburia intestinalis	Firmicutes	Clostridia	Clostridiales	Lachnospiraceae	Roseburia	-0,22	2,24E-02	4,46E-02
35,56	Alistipes spp.	Bacteroidetes	Bacteroidia	Bacteroidales	Rikenellaceae	Alistipes	0,11	2,30E-01	2,71E-01
32,82	Lachnospiraceae_NK4A136_group spp.	Firmicutes	Clostridia	Clostridiales	Lachnospiraceae	Lachnospiraceae_NK4A136_group	-0,27	4,83E-03	1,38E-02
29,62	Lachnoclostridium spp._2	Firmicutes	Clostridia	Clostridiales	Lachnospiraceae	Lachnoclostridium	-0,36	9,78E-05	1,96E-03
29,38	Bifidobacterium breve	Actinobacteria	Actinobacteria	Bifidobacteriales	Bifidobacteriaceae	Bifidobacterium	0,29	1,82E-03	1,21E-02
29,01	Blautia spp.	Firmicutes	Clostridia	Clostridiales	Lachnospiraceae	Blautia	0,1	2,92E-01	3,08E-01
28,68	Streptococcus mitis/parasanguinis	Firmicutes	Bacilli	Lactobacillales	Streptococcaceae	Streptococcus	0,18	5,54E-02	8,53E-02
28,05	Blautia spp._2	Firmicutes	Clostridia	Clostridiales	Lachnospiraceae	Blautia	-0,21	2,45E-02	4,46E-02
27,99	Bifidobacterium spp.	Actinobacteria	Actinobacteria	Bifidobacteriales	Bifidobacteriaceae	Bifidobacterium	0,13	1,80E-01	2,25E-01
27,88	Terrisporobacter spp.	Firmicutes	Clostridia	Clostridiales	Peptostrepto-coccaceae	Terrisporobacter	0,08	4,16E-01	4,16E-01
27,8	Catenisphaera spp.	Firmicutes	Erysipelotrichia	Erysipelotrichales	Erysipelotrichaceae	Catenisphaera	-0,13	1,65E-01	2,20E-01
26,98	Oscillibacter spp.	Firmicutes	Clostridia	Clostridiales	Ruminococcaceae	Oscillibacter	-0,34	2,66E-04	2,66E-03
25,79	Lachnoclostridium spp._3	Firmicutes	Clostridia	Clostridiales	Lachnospiraceae	Lachnoclostridium	-0,28	2,79E-03	1,29E-02
25,66	Prevotella_9 spp.	Bacteroidetes	Bacteroidia	Bacteroidales	Prevotellaceae	Prevotella_9	0,24	1,14E-02	2,54E-02
23,45	Ruminococcaceae_UCG-013 spp.	Firmicutes	Clostridia	Clostridiales	Ruminococcaceae	Ruminococcaceae_UCG-013	-0,17	6,88E-02	9,83E-02
23,09	Allisonella spp.	Firmicutes	Negativicutes	Selenomonadales	Veillonellaceae	Allisonella	-0,1	2,91E-01	3,08E-01
21,75	Bifidobacterium bifidum	Actinobacteria	Actinobacteria	Bifidobacteriales	Bifidobacteriaceae	Bifidobacterium	0,25	8,40E-03	2,10E-02
21,69	Christensenellaceae_R-7_group spp.	Firmicutes	Clostridia	Clostridiales	Christensenellaceae	Christensenellaceae_R-7_group	0,21	2,87E-02	4,78E-02

Supplemental table 3. Correlation top 20 microbes and peripheral IS.
